# Teres minor denervation and pathologies resulting in shoulder joint instability and rotator cuff tears: A retrospective cross-sectional MRI study

**DOI:** 10.1097/MD.0000000000037232

**Published:** 2024-02-23

**Authors:** Joo Yeon Lee, Yon-Sik Yoo, Kilhwan Shon

**Affiliations:** a Department of Radiology, Camp 9 Orthopedic Clinic, Hwaseong-si, Republic of Korea; b Department of Orthopedic Surgery, Camp 9 Orthopedic Clinic, Hwaseong-si, Republic of Korea; c Department of Ophthalmology, Gangneung Asan Hospital, Gangneung-si, Republic of Korea; d Asan Artificial Intelligence Institute, Seoul, Republic of Korea.

**Keywords:** magnetic resonance imaging, quadrilateral space syndrome, rotator cuff pathologies, shoulder instability, teres minor

## Abstract

Teres minor denervation (TMD) has gained increasing attention in recent years, particularly with the advent of magnetic resonance imaging (MRI). The potential association between TMD and shoulder instability or rotator cuff tear remains a subject of interest in the orthopedic community. In this retrospective and cross-sectional study, authors aim to investigate the potential association between TMD and shoulder instability or rotator cuff tears. Authors retrospectively analyzed MRI findings from 105 patients with TMD, focusing on rotator cuff pathologies, posterior labrocapsular complex (PLCC) tears, and posteroinferior glenohumeral joint capsule alterations. Authors assessed the association between TMD and rotator cuff and PLCC tears. For the multivariate analysis, partial proportional odds models were constructed for subscapularis (SSC) and SSP tears. Rotator cuff tears were present in 82.9% of subjects, with subscapularis (SSC) tears being the most frequent (77.1%). A significant association was observed between TMD and rotator cuff pathology (*P* = .002). PLCC tears were found in 82.3% of patients, and humeral position relative to the osseous glenoid was noted in 60% of patients with TMD. A significant association was identified between TMD and shoulder instability or labral/capsular abnormalities (*P* < .001). More than half of the cases exhibited a long tethering appearance toward the axillary neurovascular bundle on T1-weighted sagittal images. Our findings suggest that TMD is significantly associated with rotator cuff tears and shoulder instability. This study highlights the importance of identifying and treating PLCC tears in patients with TMD to address shoulder instability. Further research is needed to elucidate the role of TMD in the pathogenesis of shoulder instability and rotator cuff pathology.

## 1. Introduction

Shoulder pain is a prevalent musculoskeletal complaint, with rotator cuff pathologies and shoulder instability being the most common causes. The teres minor is a rotator cuff muscle that is crucial for maintaining glenohumeral joint stability and mobility. Teres minor denervation (TMD) is an intriguing magnetic resonance imaging (MRI) finding within the realm of shoulder pathology. However, the exact correlation between TMD and shoulder pathologies, particularly rotator cuff tears and shoulder instability, remains unclear.^[1,2]^ Furthermore, the terminology surrounding quadrilateral space syndrome (QLS) and TMD is ambiguous, which has hindered the interpretation of the existing literature.

The primary aim of this study was to explore the potential association between TMD and shoulder instability or rotator cuff tear. In doing so, authors also addressed the confusion in terminology between QLS and TMD using a literature review to provide a clearer understanding of these entities. Comprehending the relationships between TMD, rotator cuff pathologies, and shoulder instability can enhance diagnostic accuracy and guide the development of targeted treatment strategies for patients with shoulder pain.

Through a comprehensive analysis of MRI in patients with TMD and various clinical presentations, authors aimed to investigate the potential association between TMD and shoulder instability or rotator cuff tears. The present study aimed to identify any associated shoulder instability or rotator cuff abnormalities. Our findings may provide valuable insights into the role of the teres minor in shoulder stability and the complex interactions between the rotator cuff muscles in patients experiencing shoulder pain. Ultimately, this research may contribute to the development of more effective treatment strategies and a better understanding of multifaceted shoulder conditions.

## 2. Materials and methods

### 2.1. Study population

**Figure 1.**
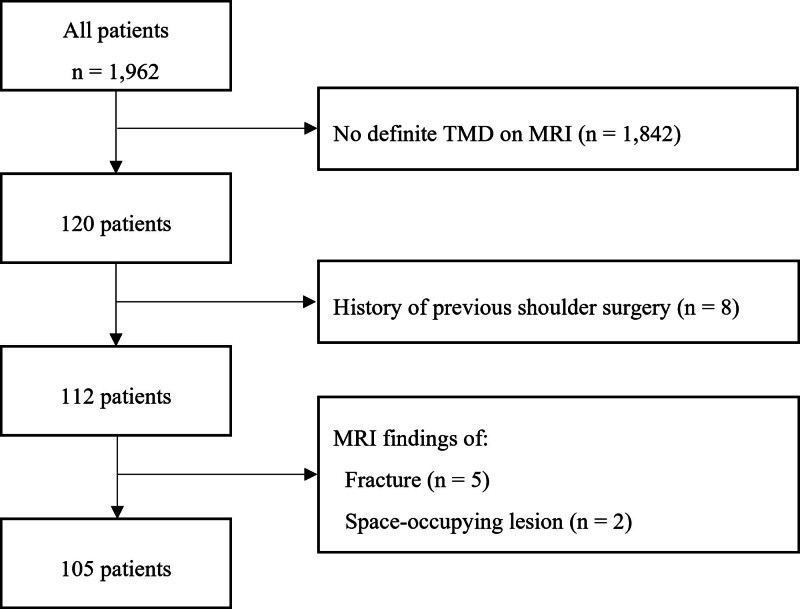
Flow chart of study participant selection and inclusion process.

A retrospective analysis was conducted on patients who underwent shoulder magnetic resonance (MR) imaging between January 01, 2016, and December 31, 2020, for various clinical reasons. Among the 1962 patients, 120 displayed teres minor denervation (TMD) and/or atrophy on MRI. Exclusion criteria included age below 18 years, a history of prior shoulder surgery, acute trauma, or imaging abnormalities such as fractures, arthritic changes, or tumors causing mass effects on the surrounding tissues. Ultimately, 105 patients met the inclusion criteria, and their MR images were reviewed (Fig. 1). The Institutional Review Board designated by the Ministry of Health and Welfare approved the study (approval no. P01-202111-21-022), and the requirement for informed consent was waived.

### 2.2. Clinical evaluation

A team of 5 orthopedic surgeons managed the clinical diagnosis and nonsurgical treatment. When required, arthroscopic surgery was performed by one of the surgeons specializing in shoulder orthopedic surgery and sports medicine. Clinical diagnosis relied on medical history and comprehensive physical examination, which included various tests and maneuvers to assess shoulder function and stability. The translation of the humeral head on the glenoid fossa was also evaluated, considering that translation greater than 1 cm was significant for all patients.

### 2.3. MRI protocol

MRI examinations were performed on patients with various clinical presentations such as shoulder pain, instability, and rotator cuff tears. All MR images were acquired using 1.5T MRI scanners following standard shoulder examination protocols, including oblique coronal fast spin-echo sequence in the plane of the long axis of the scapula, repetition time (TR)/echo time (TE) 4000/34 (effective), slice thickness 3 mm, no interslice gap, field of view (FOV) 16 cm, matrix 512 × 384, 4 number of excitations (NEX), echo train length (ETL) 9 to 14; oblique coronal with frequency-selective fat suppression (Chemsat; General Electric Medical Systems, Milwaukee, WI), TR/TE 4000/80 (effective), slice thickness 3 mm with no interslice gap, FOV 16 cm, 256 × 224 matrix, 4 NEX, ETL 8 to 10; oblique sagittal FSE, TR/TE 3500 to 4000/34 (effective), 3 mm slice thickness, skip 0.3 mm, FOV 16 cm, 512 × 224, 2 NEX; and axial FSE TR/TE 4000/34 (effective), slice thickness 3.5 mm with no interslice gap, FOV 16 cm, 512 × 384 matrix, 4 NEX, ETL 8. Two experienced musculoskeletal imaging readers, blinded to the clinical data, independently reviewed and analyzed the MR images. Any discrepancies were resolved by consensus.

### 2.4. Image analysis

#### 2.4..1. TMD assessment.

According to the original paper, with the posterior approach, the boundaries of the quadrilateral space are defined by the teres minor superiorly, capsule of the GH joint and humerus laterally, long head of the triceps brachii medially, and fascia and adipose tissue inferiorly.^[3]^ TMD was determined by examining the muscle for atrophy, fatty infiltration, or increased T2 signal intensity. The Goutallier classification system was used to grade fatty infiltration. In cases of denervation, the extent and severity of the changes were documented.^[4–6]^

#### 2.4..2. Evaluation of associated rotator cuff pathologies.

For patients with TMD, a comprehensive assessment of the rotator cuff tendons, labrum, and glenohumeral joint capsule was performed to identify any related pathologies. Documented rotator cuff pathologies include tendinopathy, partial-thickness tears, and full-thickness tears of the SSP, infraspinatus (ISP), and subscapularis (SSC) tendons.

#### 2.4..3. Assessment of shoulder instability, posterior labrocapsular complex (PLCC), and humeral position.

Shoulder instability was evaluated by examining labral tears using specific criteria. Humeral position relative to the glenoid fossa was assessed using a measurement method, reporting a negative value or relative posterior position of the humeral head if the center was posterior to the reference line.^[7]^

#### 2.4..4. Glenohumeral joint capsule evaluation

**Figure 2.**
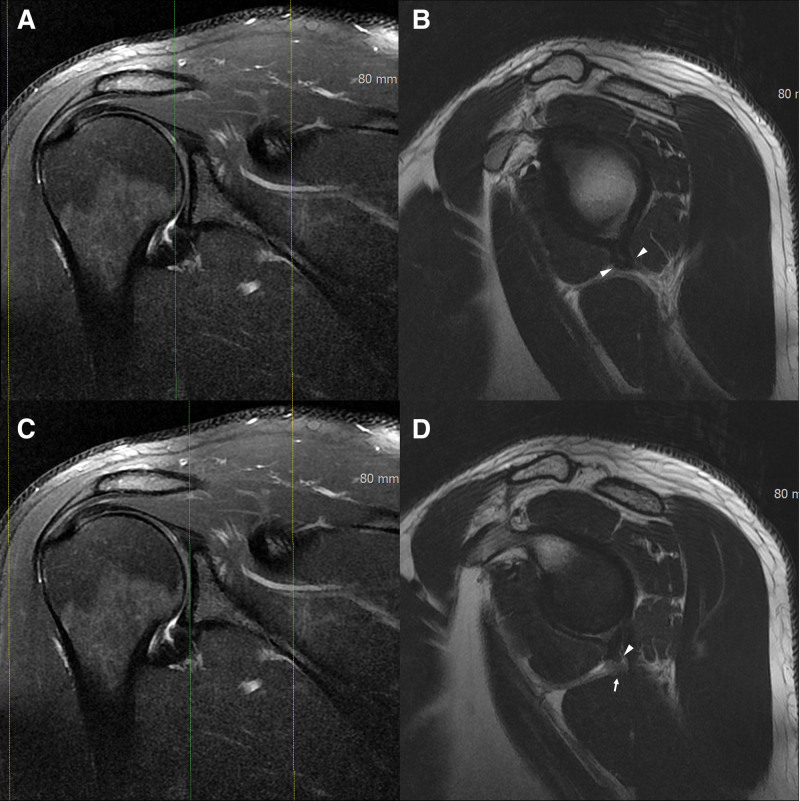
A. A fat-saturated T2-weighted (FST2W) oblique coronal image of a 28-year-old male patient experiencing vague posterior shoulder pain for years illustrates joint capsule thickening. The two yellow scout lines represent the range of sagittal images obtained, with the green line indicating the location of the sagittal image shown in B. B. A T1-weighted (T1W) oblique sagittal image displays a redundant posteroinferior joint capsule. C. The same oblique coronal FST2W, with the green line shifted 3.3 mm medially, is presented to demonstrate that the two T1W oblique sagittal images in B and D are consecutive. D. A T1WI oblique sagittal image taken 3.3 mm medial to Figure B demonstrates the protrusion of the joint capsule and the obliteration of the fat plane between the capsule and the axillary nerve, potentially indicative of a fibrous band associated with quadrilateral space syndrome.

The glenohumeral joint capsule was assessed for abnormalities, particularly at the posteroinferior border, which is a quadrilateral boundary. Notably, most subjects demonstrated noticeable changes at the posteroinferior border irrespective of the presence or absence of other rotator cuff pathologies (Fig. 2). This observation suggests a potential association between TMD and joint capsule redundancy at the posteroinferior border, which may explain the “fibrous band” observed in quadrilateral space syndrome.

Based on this evaluation, the capsule contours were categorized into 4 types. Type 1a represents severe redundancy and thickening of the posteroinferior joint capsule, obliterating the fat plane of the axillary neurovascular bundle in the quadrilateral space. Tethering peaks toward the axillary neurovascular bundle were designated as type 1b when taller than wide and type 1c when small and pointing without direct abutting. Type 1d describes severe redundancy without peaks.

Currently, there is limited evidence regarding the characteristics of normal joint capsules and whether this observed redundancy in the glenohumeral joint capsule is consistently observed in the general population or is specifically associated with TMD, shoulder instability, or rotator cuff tears. Our evaluation was primarily descriptive because of the scarcity of available evidence. Based on previous reports on the plastic deformity of joint capsules, authors can presume that these findings might be more commonly observed in patients with shoulder pain.

### 2.5. Statistical analysis

Descriptive statistics were calculated, and inter-observer agreement was determined using the intraclass correlation coefficient (ICC). The chi-square test was employed to evaluate the impact of TMD on other rotator cuff pathologies. For multivariate analysis, partial proportional odds models were constructed for SSC and SSP tears using a stepwise method with a significance threshold of *P* < .10.^[8]^ Because of the zero-frequency cells, models for the ISP and teres minor tears were not built. In this study, missing data was not an issue as our dataset was complete. SAS software version 9.4 was used for all the statistical analyses.

## 3. Results

### 3.1. Study population characteristics

The study included 105 patients, with a mean age of 48.0 years and a predominantly male population (78.1%). The most frequent initial clinical impressions were frozen shoulder (29.5%), impingement syndrome (27.6%), and shoulder instability (21.0%). QLS was suspected in 11 patients (10.5%) (Table 1).

**Table 1:** Demographics and clinical characteristics.

Age, years (SD)	48.0 (12.4)
Female, count	23 (21.9)
Right, count	59 (56.2)
Frozen shoulder, count	31 (29.5)
Impingement, count	29 (27.6)
Shoulder instability, count	22 (21.0)
QLS, count	11 (10.5)
Other conditions (etc.), count	12 (11.4)

Numbers in the parentheses are percentages if not denoted otherwise.

QLS = Quadrilateral Space Syndrome, SD = standard deviation.

Inter-observer agreement was good for assessing deltoid atrophy (ICC 0.83), TMD grading (ICC 0.79), and rotator cuff tears (ICC 0.78–0.88). However, it was lower for evaluating the relative posterior position of the humeral head (ICC 0.66) and poor for determining the presence of PLCC tears (ICC 0.23) (Table 2).

**Table 2:** Inter-observer agreement of magnetic resonance image interpretation.

	ICC	95% CI
Combined deltoid atrophy	0.83	0.76–0.88
Grading of teres minor denervation	0.79	0.70–0.85
Posterior labrocapsular complex tear	0.43	0.27–0.61
Relative posterior position of the humeral head	0.66	0.54–0.76
Subscapularis tear grading	0.78	0.69–0.85
Supraspinatus tear grading	0.80	0.71–0.86
Infraspinatus tear grading	0.88	0.82–0.91
Teres minor tear grading	0.81	0.74–0.87

CI = confidence interval, ICC = intraclass correlation coefficient.

### 3.2. Rotator cuff pathologies in TMD

Rotator cuff tears were present in 82.9% of the subjects, with SSC tears being the most frequent (77.1%), followed by SSP (59.0%), ISP (24.8%), and teres minor (6.7%). A significant association was observed between TMD and rotator cuff pathology (*P* = .002). Additionally, the severity of teres minor atrophy was significantly correlated with individual rotator cuff tears (Table 3).

**Table 3:** Frequency of associated findings according to the teres minor denervation grade.

	Teres minor denervation grade			*P* value
	0 (n = 16)	1 (n = 53)	2 (n = 36)	
Shoulder joint pathology				
Posterior labrocapsular complex tear	11 (69)	46 (87)	30 (83)	.244
Relative posterior humeral position	8 (50)	30 (57)	25 (69)	.323
Rotator cuff tears				
Intact RC	3 (19)	12 (23)	3 (8)	.210
Any tear of any RC muscle	13 (81)	41 (77)	33 (92)	
SSCT				
No tear	4 (25)	16 (30)	5 (14)	.037*
<50%	10 (63)	30 (57)	17 (47)	
≥50%	2 (13)	7 (13)	14 (39)	
SST				
No tear	8 (50)	28 (53)	7 (19)	.004*
<50%	2 (13)	16 (30)	11 (31)	
≥50%	6 (38)	9 (17)	18 (50)	
IST				
No tear	14 (88)	44 (83)	21 (58)	.037*
<50%	0 (0)	5 (9)	5 (14)	
≥50%	2 (13)	4 (8)	10 (28)	
TM				
No tear	16 (100)	52 (98)	30 (83)	.012*
<50%	0 (0)	1 (2)	6 (17)	
≥50%	0 (0)	0 (0)	0 (0)	

Please note that the numbers in the table represent the frequency of the specific characteristics, and the numbers in parentheses represent the percentages of each category.

IST = infraspinatus tendon, RC = rotator cuff, SSCT = subscapularis tendon, SST = supraspinatus tendon, TM = Teres Minor Tendon.

* Indicates statistical significance (*P* < .05).

### 3.3. PLCC tear and relative humeral position to the osseous glenoid in TMD

PLCC tears were found in 87 (82.3%) patients, and humeral position relative to the osseous glenoid was noted in 63 (60%) patients with TMD. A significant association was identified between TMD and shoulder instability or labral/capsular abnormalities (*P* < .001).

### 3.4. Posteroinferior glenohumeral joint capsule in TMD

More than half of the cases (51.4%) exhibited a long tethering appearance toward the axillary neurovascular bundle on T1-weighted sagittal images (type 1b). In 2.9% of the cases, complete obliteration of the fat plane with broad abutting to the axillary neurovascular bundle was observed (type 1a). Type 1c, characterized by a small peak, and type 1d, featuring severe smooth redundancy, were also commonly observed, occurring in 31.4% and 14.3% of cases, respectively.

### 3.5. Association between TMD and severity of rotator cuff tear

Two multivariate models using the partial proportional odds model were employed to assess the relationship between TMD grade and SSC and SSP tear severities. The SSC model revealed a strong association between tear severity and age, with the odds of increased tear severity being 1.14 (95% CI 1.09–1.20; *P* < .001) per additional year of age. The TMD grade was not significant and was removed during the elimination.

The SSP model demonstrated a significant positive association between tear severity and age (OR, 1.22; 95% CI 1.14–1.31; *P* < .001). The negative association with female sex (OR, 0.34; 95% CI 0.10–1.15; *P* = .083) was not statistically significant. Importantly, supraspinatus tears exceeding 50% thickness showed a significant negative association with TMD for both grade 1 (OR, 0.05; 95% CI 0.01–0.28; *P* = .001) and grade 2 (OR 0.16; 95% CI 0.03–0.90; *P* = .038) (Table 4).

**Table 4:** Partial proportional odds model for subscapularis and supraspinatus tears.

	OR estimate	95% CI	*P* value
Subscapularis model			
Age	1.14	1.09-1.20	<.001*
Supraspinatus model			
Age	1.22	1.14-1.30	<.001*
Female	0.34	0.10-1.15	.083
Supraspinatus tear < 50%			
Reres minor denervation grade 1	0.29	0.07-1.26	.099
Teres minor denervation grade 2	0.97	0.19-5.00	.968
Supraspinatus tear ≥ 50%			
Teres minor denervation grade 1	0.05	0.01-0.28	.001*
Teres minor denervation grade 2	0.16	0.03-0.90	.038*

The table presents the results of the partial proportional odds models for subscapularis and supraspinatus tears. The OR estimate, 95% CI, and *P* value columns provide the corresponding odds ratio estimates, confidence intervals, and p-values for each variable in the models.

OR = odds ratio, CI = confidence interval.

* Indicates statistical significance (*P* < .05).

## 4. Discussion

In this study, our primary focus was on the potential association between TMD and shoulder instability or rotator cuff tears. Authors aimed to investigate the possible impact of TMD on the balance of rotator cuff muscles and its association with subsequent tears. Research suggests that isolated TMD is relatively uncommon (2.4%), although not rare, and often co-occurs with concomitant rotator cuff tears.^[5,9]^ The SSC tendon was the most frequently involved tendon, whereas the teres minor tendon was the least affected.

Authors observed a high prevalence of PLCC tears in patients with TMD, indicating a possible association between shoulder instability and TMD. PLCC plays a crucial role in maintaining shoulder stability by resisting excessive translation and providing proprioceptive feedback, and its damage may lead to instability. Furthermore, our results showed a significant association between the severity of rotator cuff tears and TMD severity in univariate analysis. However, after the multivariate analysis, this association was mainly attributed to age.

**Figure 3.**
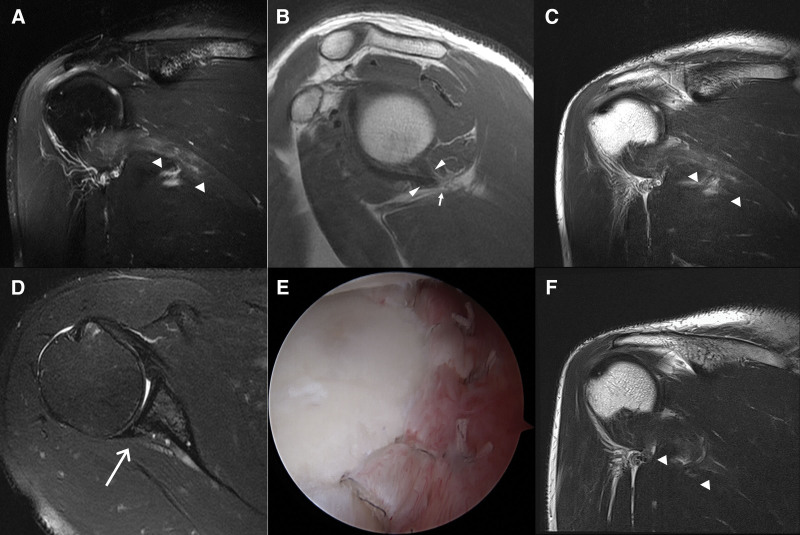
Preoperative and postoperative MRI images of a 42-year-old male patient with right shoulder vague pain. The patient’s shoulder pain began 5 years ago, and the pain aggravated 1 month ago with night pain. Physical examination reveals full passive ROM, positive abduction and external rotation provocation test, and tenderness over the quadrilateral space. The patient underwent arthroscopic labral repair, and the symptoms significantly improved by the 4-week follow-up visit. A. Preoperative fat-saturated T2-weighted (FST2W) oblique coronal image demonstrates denervation edema of the teres minor (short arrowheads) and a small subacromial-subdeltoid bursitis. B. Oblique sagittal T1-weighted image (T1WI) reveals decreased volume of the teres minor and the posteroinferior joint capsule tethered (long arrowheads) to the axillary neurovascular bundle (short arrow). C. Preoperative T2-weighted (T2W) oblique coronal image displays denervation edema mixed with fatty infiltration. D. Preoperative FST2W axial image exhibits a posterior labrocapsular complex tear (long arrow). E. Arthroscopic image presents satisfactory labral repair. F. Postoperative T2-weighted oblique coronal image obtained 4 weeks after the arthroscopic surgery shows decreased high signal intensity of the teres minor compared to that of Figure C.

**Figure 4.**
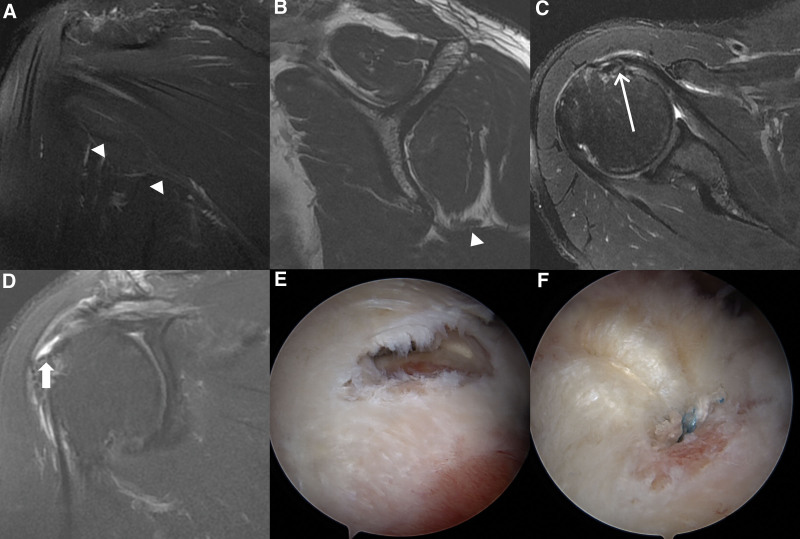
A 62-year-old male patient presented with recent limited ability to move his right arm and has been experiencing poorly localized pain in the posterior shoulder region for decades. The patient underwent arthroscopic rotator cuff repair and subsequent rehabilitation. At a 30-month follow-up, the patient was symptom-free. A. On fat-saturated T2-weighted (FST2W) oblique coronal image, decreased volume of the teres minor is suspected with minimal denervation edema (arrowheads). B. Oblique sagittal T1WI shows severe atrophy of the teres minor (arrowhead). C. Axial FST2W image reveals an articular side partial-thickness tear of the subscapularis and mild medial subluxation of the long head of the biceps tendon (long arrow). D. On FST2W oblique coronal image, a full-thickness tear of the supraspinatus is observed. E. Arthroscopic image confirms the full-thickness tear of the supraspinatus. F. Satisfactory arthroscopic rotator cuff repair is performed.

The ambiguity between the QLS and TMD may stem from differences in clinical approaches and technological advancements since the original QLS paper in 1967.^[3]^ TMD, detected using MRI, became possible only with the commercial availability of MRI machines in the 1980s.^[5,6,10–12]^ Although the precise relationship between TMD and QLS in the presence of other rotator cuff pathologies remains unclear, some patients with TMD and concomitant rotator cuff tears have reported improvements in vague posterior shoulder pain following less invasive interventions, such as arthroscopic labral repair or rotator cuff repair (Figs. 3 and 4). These procedures are typically complemented by physiotherapy and targeted rehabilitation efforts to strengthen the posterior rotator cuff muscles. In this context, posterior muscle strengthening may also be beneficial in addressing posterior shoulder instability related to PLCC tears, along with TMD and concomitant rotator cuff pathologies.^[12,13]^ Further research is needed to identify the most effective interventions for TMD and related pathologies, including surgical and nonsurgical approaches.

This study has several limitations that need to be acknowledged. First, the retrospective design of the study may introduce potential selection bias. Considering that our study focuses on patients with TMD, there may be an inherent bias in the patient population, as those without TMD were not included for comparison. Second, the sample size may not be sufficiently large to draw definitive conclusions, and future studies with larger sample sizes are needed to validate our findings. Finally, as authors focused on the MR findings, the clinical significance of the observed associations remains to be determined. Future prospective studies incorporating both imaging and clinical data are needed to further elucidate the role of TMD in the pathogenesis of shoulder instability and rotator cuff pathology.

Our findings may enhance our understanding of the role of the teres minor in maintaining shoulder stability and the complex interactions between the rotator cuff muscles in patients presenting with shoulder pain. Clinicians should be aware of the potential association between TMD and rotator cuff muscle imbalance, which can lead to consecutive rotator cuff tears. Additionally, our study highlights the importance of identifying and treating PLCC tears in patients with TMD to address shoulder instability. These findings may help guide diagnosis and treatment strategies for patients with shoulder pain.

## 5. Conclusion

Our study provides valuable insights into the potential association between teres minor denervation and shoulder instability or other rotator cuff pathologies. Our findings suggest that teres minor denervation may significantly affect the balance of the rotator cuff muscles, potentially leading to subsequent tears and shoulder instability. Furthermore, authors found a high prevalence of PLCC tears and joint capsule redundancy in patients with TMD, highlighting the importance of thorough examination of the glenohumeral joint capsule in such patients. These results underscore the need for a comprehensive assessment of teres minor and its potential role in shoulder instability and rotator cuff pathologies. Further research is warranted to better understand the complex relationship between teres minor denervation, shoulder instability, and rotator cuff pathologies and to identify the most effective interventions for managing these conditions.

## Author contributions

**Conceptualization:** Joo Yeon Lee, Yon-Sik Yoo.

**Formal analysis:** Kilhwan Shon.

**Investigation:** Joo Yeon Lee.

**Methodology:** Kilhwan Shon.

**Resources:** Yon-Sik Yoo.

**Software:** Kilhwan Shon.

**Supervision:** Yon-Sik Yoo.

**Writing – original draft:** Joo Yeon Lee.

**Writing – review & editing:** Joo Yeon Lee.
